# Ovarian hormones attenuate right ventricular remodeling in a rat model of pulmonary arterial hypertension

**DOI:** 10.1007/s10237-025-02033-7

**Published:** 2025-12-17

**Authors:** Becky A. Hardie, Jessica Huberts, Michael Bennington, Daniela Valdez-Jasso

**Affiliations:** Department of Bioengineering, University of California, San Diego, La Jolla, CA USA

**Keywords:** Pulmonary arterial hypertension, Sugen–hypoxia, Right ventricular diastolic function, Ovarian hormones, Mechanical properties

## Abstract

Pulmonary arterial hypertension (PAH) induces chronic pressure overload on the right ventricle (RV), leading to progressive remodeling and eventual failure. While PAH is more prevalent in women overall, men and postmenopausal women have worse clinical outcomes. Here, we investigated how sex and ovarian hormones influence RV remodeling during the progression of PAH. Using the sugen–hypoxia (SuHx) rat model, we assessed RV hemodynamics, tissue mechanics, and collagen composition in male, ovary-intact female, and ovariectomized (OVX) female rats across four disease stages. While all three groups experienced elevated pulmonary and ventricular pressures and rapidly responded with hypertrophy and stiffening, RV remodeling progressed differently in the absence of ovarian hormones. Male and OVX rats exhibited marked increases in end-diastolic pressure and myocardial stiffness, as well as higher chamber elastances. Ovary-intact female rats largely preserved diastolic function with milder stiffening. Collagen accumulation was observed in all groups, but only male and OVX rats exhibited significant elevations in pyridinoline cross-linking—aligning with the most severe additional mechanical changes, namely increased passive stiffness. This suggests that ovarian hormones moderate the severity of SuHx-induced RV remodeling by limiting myocardial stiffening and collagen cross-linking. These findings emphasize the need to consider sex and hormonal status in preclinical PAH research and suggest that extracellular matrix cross-linking may be a targetable contributor to maladaptive right heart remodeling.

## Introduction

Pulmonary arterial hypertension (PAH) is a progressive vasculopathy marked by sustained vascular remodeling and elevated mean pulmonary arterial pressure (mPAP) above 20 mmHg (Simonneau et al. 2019). The resulting increase in pulmonary vascular resistance places a chronic pressure overload on the right ventricle (RV), ultimately leading to adverse RV remodeling and failure. Although PAH is more prevalent in women, with registries reporting a two–fourfold higher incidence compared to men (Jacobs et al. 2014; Ventetuolo et al. 2014), pre-menopausal women tend to have more preserved RV function and improved survival compared to postmenopausal women and men (Ventetuolo et al. 2011). Both systolic and diastolic RV function are impaired in PAH, but diastolic dysfunction has emerged as a particularly important predictor of adverse outcomes (Rain et al. 2013; Trip et al. 2015). Despite this, the relationship between sex and diastolic dysfunction in PAH remains poorly understood. While sex differences in disease incidence and survival are well-documented, the relationship between sex and the mechanical and structural remodeling of the RV is less clear. Most preclinical studies have relied exclusively on male subjects. However, recent work by Kwan et al. (2024) using the sugen–hypoxia (SuHx) rat model of PAH suggested that RVs from male and female rats undergo distinct forms of remodeling in response to pressure overload. Notably, male rats exhibited a greater increase in end-diastolic (ED) elastance—a measure of chamber stiffness—and their elevated end-diastolic pressure was primarily attributed to predicted myocardial stiffening. In contrast, ovary-intact females showed milder changes to ED elastance, with ED pressure increases largely explained by geometric chamber remodeling. Ovariectomized (OVX) females exhibited an intermediate phenotype, combining the predicted stiffening of the male response with the hypertrophy-dominated ovary-intact female remodeling. Other studies, such as those by Kakaletsis et al. (2023), have characterized RV stiffening in male animals as a multifaceted process involving wall thickening, collagen accumulation, and myocardial stiffening, with passive stiffness being the dominant contributor to diastolic dysfunction. Cheng et al. (2022) reported sex-dependent collagen accumulation and hormone-dependent collagen organization in hypertensive RVs, while diastolic function remained similar across groups. This complements prior work and highlights the need to clarify the contributions of ovarian hormone to RV mechanical remodeling. To address these gaps and validate the model predictions of Kwan et al. (2024), we conducted a comprehensive study examining how sex and ovarian hormone status influence RV remodeling during the progression of pulmonary hypertension. We assessed RV function and structure across four experimental stages in male, ovary-intact female, and ovariectomized female rats using the SuHx model of PAH, expanding on Kwan et al. (2024)’s study by including one timepoint before and one timepoint after their data collection and model predictions. We obtained in vivo hemodynamic measurements prior to ex vivo mechanical testing and collagen analyses to characterize group- and stage-dependent changes in RV diastolic function and passive stiffness. We hypothesized that male SuHx rats would experience the most severe diastolic dysfunction, driven by substantial myocardial stiffening and collagen accumulation. In contrast, we expected ovary-intact females to exhibit milder diastolic impairment, with remodeling dominated by hypertrophy rather than tissue stiffening. OVX females were anticipated to show an intermediate phenotype, reflecting features of both the male and ovary-intact female RV remodeling profiles.

## Methods

### Rat model of pulmonary arterial hypertension

All procedures were approved by the Institutional Animal Care and Use Committee at University of California, San Diego, and carried out in accordance with the established regulations. We used the sugen–hypoxia (SuHx) rat model of pulmonary arterial hypertension (PAH), which closely mimics the plexiform lesions found in human PAH explants (Abe et al. 2010). Sugen (SU5416 S8442 MilliporeSigma, CAS Number 204005-46-9, PubChem Substance ID 24278606 Sigma-Aldrich, MO, USA), a vascular endothelial growth factor receptor 2 inhibitor, was dissolved in dimethylsulfoxide (DMSO) and filtered through a 0.2 $\mu$m syringe filter before administration.

**Table 1:** Number of rats from each group included in each experiment

Group	Experimental Stage	Biaxial Testing & Morphology (Figs. 4–7)	Hemodynamic Measurements (Figs. 2–3)	Hydroxyproline Collagen Content (Fig. 8a)	PYD Collagen Cross-linking (Fig. 8b)
64 Males	Control	19	18	10	3
	SuHx Week 4	16	14	10	4
	SuHx Week 8	16	15	10	4
	SuHx Week 12	13	11	10	4
65 Intact Females	Control	20	19	12	3
	SuHx Week 4	14	14	10	3
	SuHx Week 8	20	17	10	3
	SuHx Week 12	11	11	9	4
56 OVX Females	Control	17	17	13	3
	SuHx Week 4	12	11	7	3
	SuHx Week 8	13	11	9	3
	SuHx Week 12	14	13	8	4

The rats counted in each column are also included in all the columns to the left (i.e., all the rats which had hydroxyproline collagen content measured also have hemodynamic measurements, morphological data, and mechanical data)

Sprague Dawley rats (Charles River, Hollister, CA, USA) were randomly assigned to a control or SuHx group. Male (7 weeks old, 228 g) and ovary-intact female (8 weeks old, 190 g) rats received a single subcutaneous injection of sugen (20 mg/kg) in the posterior of the neck and were housed in $10\%\,\hbox {O}_{2}$ normobaric hypoxia chambers (ProOx E702, Biospherix, NY, USA). Following three weeks of hypoxia, rats were returned to normoxia ($21\%\,\hbox {O}_{2}$) for 1, 5, or 9 weeks, corresponding to 4, 8, and 12 weeks of SuHx exposure, respectively. An additional group of female rats underwent ovariectomy (OVX; $\approx$ 7 weeks old, 228 g) at Charles River Laboratories approximately 10 days prior to SuHx induction to allow time for incision healing and hormone clearance (Dougherty et al. 2017; Woolley and McEwen 1993). Age-matched controls were not injected, kept in normoxia throughout, and combined into three (male, female, OVX) control groups for analysis (Kwan et al. 2021). In total, 185 rats were included in this study (Table 1).

### In vivo pressure–volume measurements

Terminal open-chest surgery and right heart catheterization were performed to acquire in vivo hemodynamic measurements and confirm hypertension as described previously (Vélez-Rendón et al. 2018). Briefly, animals were anesthetized and underwent tracheotomy and thoracotomy under continuous administration of oxygen mixed with 2.5% isoflurane. A 1.9 French admittance catheter (Transonic Scisense, ON, CA) was inserted into the right ventricle to record pressure–volume time series waveforms (LabChart Pro v8.1.30, ADInstruments Inc., CO, USA). Following steady-state pressure–volume measurements, caval occlusions were performed to investigate the effect of changes in preload. The RV was punctured a second time near the outflow tract to insert a 1.6 French dual pressure sensor catheter (Transonic Scisense, ON, CA), which was advanced past the pulmonic valve to simultaneously measure RV and main pulmonary artery pressures to determine mean pulmonary arterial pressure (mPAP) and confirm pulmonary hypertension (mPAP > 20 mmHg).

### Hemodynamic data processing

Pressure–volume waveforms were analyzed to identify repeatable steady-state sections. A custom-written MATLAB script (R2024a, The MathWorks Inc.) was used to align pressure and volume traces across three consecutive heartbeats, interpolated throughout the cardiac cycle, and averaged to generate representative loops. The point of maximum pressure-to-volume ratio defined end systole (ES), while the point of minimum pressure during the smallest volume change defined end diastole (ED). The lowest steady-state pressure was normalized to 0 mmHg and used to adjust ES and ED pressures. Stroke volume (SV) and ejection fraction (EF) were calculated using the ES and ED volumes as $SV = EDV - ESV$ and $EF = (SV/EDV)\times 100$. Pressures, volumes, and ejection fraction were averaged per animal across multiple waveform regions. Elastances were computed from end-systolic and end-diastolic pressure–volume relations during vena cava occlusion (Appendix Fig. 9), with up to 40% reduction in ES pressure and matched steady-state ED volume (Vélez-Rendón et al. 2018). ES elastance, a measure of ventricular contractility, was defined as the slope of the best-fit line through the ES points; ED elastance was obtained by fitting $P(V) = \gamma e^{\beta V}$ and calculating chamber stiffness $(dP/dV = \gamma \beta e^{\beta V})$ at the steady-state ED volume.

### Ex vivo tissue preparation and biaxial testing

**Fig. 1:**
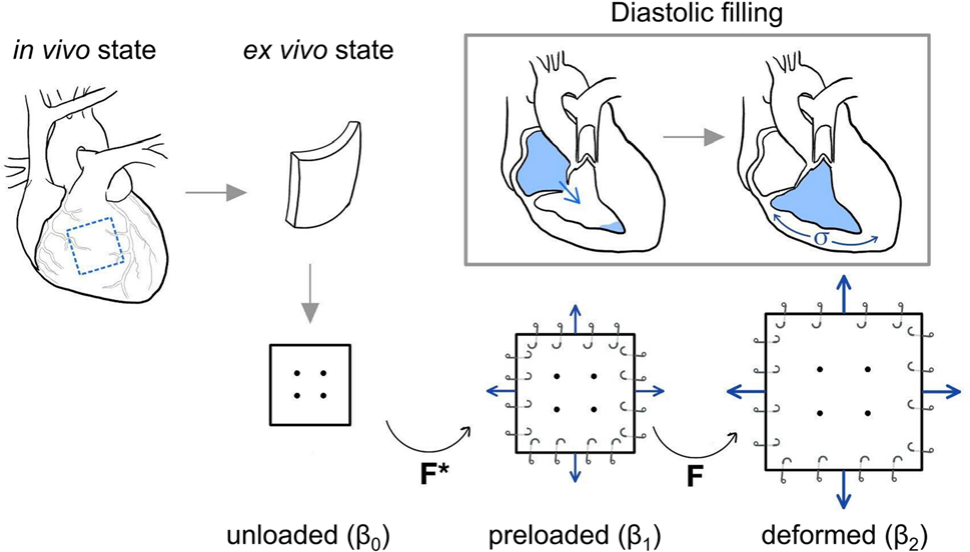
Graphical representation of the right ventricle in situ, after being isolated and cut into a square for mechanical testing (ex vivo unloaded state), and when assembled and loaded onto the planar biax device (preloaded & deformed states). The biaxial cyclic loading and unloading imposed on the sample mimic the diastolic filling and relaxation phases of the cardiac cycle. The mapping from the unloaded to deformed tissue states is done using the deformation gradients $\mathbf {F^*}$ and ${\textbf{F}}$

Following hemodynamic measurements, the animals were exsanguinated, and their hearts were flushed with cold phosphate-buffered saline (PBS; pH 7.4; 137 mM NaCl, 2.7 mM KCl, 1.8mMKH_2_ PO_4_, 10mMNa_2_ HPO_4_) containing heparin (USP 5000 units/mL, MWI Veterinary Supply, USA, cat #054255). The RV was immediately isolated and cut into a square aligned along the apex-outflow tract (AOT) direction ($x_1$) and perpendicular ($x_2$) circumferential axis (Fig. 1). Sample dimensions (average ex vivo side length: $8.23 \pm 1.02$ mm) and five thickness values were measured using a digital caliper (Absolute Digimatic Caliper, Mitutoyo, USA). Four graphite markers were placed on the epicardial surface in the middle of the sample to track tissue deformation.

Four custom-made hooks secured the sample to the planar biaxial testing system (ElectroForce Planar Biaxial TestBench Instrument, TA Instruments - Electroforce, MN, USA). The samples remained submerged in 37°C PBS throughout the tests. Samples were preloaded to 3.0 ± 0.2 grams to ensure planar displacement in the $x_1$ and $x_2$ directions. Preconditioning involved two sets of equibiaxial stretch: 8%, 10%, and 12% of the ex vivo side length at 0.2 Hz. The mechanical test consisted of seven blocks of displacement-controlled loading (15 triangle-waveform cycles at 0.5 Hz) with $x_1:x_2$ ratios of 1:1, 1:0.5, 1:0.25, 1:1, 0.5:1, 0.25:1, and 1:1, reaching up to 10% stretch.

### Mechanical data processing

Biaxial loads and pixel coordinates of the epicardial marker positions were analyzed in Python (Google Colab, Python 3). The markers were tracked at 200 Hz (Prosilica GE, Allied Vision), and the loads were recorded at 20 Hz (ElectroForce 1000 g, TA Instruments). Load measurements were upsampled to 200 Hz via cubic interpolation to synchronize with the marker locations. Data were smoothed using a Savitzky–Golay filter and the final three loading cycles of each block were averaged to compute stress and strain (Vélez-Rendón et al. 2019).

The deformation gradient ${\textbf{F}}$ was calculated via isoparametric mapping and used to compute the Green strain tensor ${\textbf{E}} = \frac{1}{2}({\textbf{F}}^{T}{\textbf{F}}-{\textbf{I}})$. First Piola–Kirchhoff stress ${\textbf{P}}$ was derived from the undeformed cross-sectional area (*A*) and the current measured load converted to force (*f*) as ${\textbf{P}} = \frac{f}{A}$. Second Piola–Kirchhoff stress was computed as ${\textbf{S}} = {\textbf{F}}^{-1}{\textbf{P}}$.

### Constitutive modeling

Given that the tissue was not truly unloaded at any point during the mechanical test, we introduced a preloaded configuration $\beta _1$ (Fig. 1). Here, $\beta _0$ represents the theoretical zero-stress state, $\beta _1$ the preloaded experimental reference state, and $\beta _2$ the set of experimentally stretched states. A deformation gradient $\mathbf {F^*}$ maps $\beta _0 \rightarrow \beta _1$, and ${\textbf{F}}$ maps $\beta _1 \rightarrow \beta _2$, giving ${\hat{\textbf{F}}} = \mathbf {FF^*}$. Pre-stretches ($\Lambda ^{*}_{1}$ and $\Lambda ^*_2$), the diagonal components of $\mathbf {F^*}$, were calculated from measured stresses ($S_{11}$, $S_{22}$), assuming linear deformation between $\beta _0$ (zero-stress), $\beta _1$ (preloaded), and $\beta _2$ (initial deformed state):

1 $$\Lambda ^{*}_{1} = \sqrt{\frac{S_{11}\Lambda _2}{\Lambda _1}+1}\text { , } \Lambda ^*_2 = \sqrt{\frac{S_{22}\Lambda _1}{\Lambda _2}+1}.$$

The Green strain tensor that represents the entire deformation from $\beta _0$ to $\beta _2$ was then expressed as $\hat{\textbf{E}} = \frac{1}{2}(\hat{\textbf{F}}^{T}\hat{\textbf{F}}-{\textbf{I}})$. The shear components of ${\hat{\textbf{E}}}$ were negligible in all test specimens, so only the normal apex-outflow tract and circumferential components $\hat{E}_{11}$ and $\hat{E}_{22}$ were analyzed and formulated in terms of $\Lambda$ as:

2 $$\begin{aligned} \hat{E}_{11} = \frac{1}{2}({{\Lambda ^*_1}^2\Lambda _1^2-1}) \text { , } \hat{E}_{22} = \frac{1}{2}({{\Lambda ^*_2}^2\Lambda _2^2-1}). \end{aligned}$$

Assuming an incompressible planar hyperelastic material, a Fung-type exponential strain-energy function is:

3 $$\begin{aligned} W({\hat{\textbf{E}}}) = C(e^{Q({\hat{\textbf{E})}}} - 1) \end{aligned}$$

where

4 $$\begin{aligned} Q({\hat{\textbf{E}}})= \hat{a}_1\hat{E}_{11}^2 + \hat{a}_2\hat{E}_{22}^2 + \hat{a}_3\hat{E}_{11}\hat{E}_{22}. \end{aligned}$$

Stress with respect to $\beta _0$ is

5 $$\begin{aligned} {\hat{\textbf{S}}} = C\frac{\partial e^{Q({\hat{\textbf{E}}})}}{\partial {\hat{\textbf{E}}}} \frac{\partial {\hat{\textbf{E}}}}{\partial \Lambda }, \end{aligned}$$

with components:

$$\begin{aligned} \hat{S}_{{11}} = & C{\Lambda ^*_1}^{2} e^{{Q(\Lambda )}} \left(\hat{a}_{1} ({\Lambda ^*_1}^{2} \Lambda _{1}^{2} - 1)+ \frac{{\hat{a}_{3} }}{2}({\Lambda ^*_2}^{2} \Lambda _{2}^{2} - 1)\right)\Lambda _{1} \\ \end{aligned}$$

$$\begin{aligned} \hat{S}_{22}= & C{\Lambda ^*_2}^2e^{Q(\Lambda )}\left(\hat{a}_2({\Lambda ^*_2}^2\Lambda _2^2-1)+\frac{\hat{a}_3}{2}({\Lambda ^*_1}^2\Lambda _1^2-1)\right)\Lambda _2 \end{aligned}$$

$$\begin{aligned} \hat{S}_{12} = {\textbf{0}} \end{aligned}$$

6 $$\begin{aligned} \hat{S}_{21} = {\textbf{0}} \end{aligned}$$

where

7 $$\begin{aligned} Q(\Lambda ) = \frac{1}{4} \left(\hat{a}_1({\Lambda ^*_1}^2\Lambda _1^2-1)^2 + \hat{a}_2({\Lambda ^*_2}^2\Lambda _2^2-1)^2 \nonumber+ \hat{a}_3({\Lambda ^*_1}^2\Lambda _1^2-1)({\Lambda ^*_2}^2\Lambda _2^2-1)\right). \end{aligned}$$

The model parameters $\theta = \{C, \hat{a}_1, \hat{a}_2, \hat{a}_3\}$ were optimized using the limited-memory Broyden–Fletcher–Goldfarb–Shanno (L-BFGS) algorithm, which minimized an objective function consisting of the squared difference between experimental stress ${\textbf{S}}(t_j)$ and model stress ${\hat{\textbf{S}}}(t_j, \theta , {\Lambda ^*_1}, {\Lambda ^*_2})$:

8 $$\begin{aligned} \phi (\theta ) = \sum _i^2 \sum _j^n \left( S_{ii}(t_j) - \hat{S}_{ii}(t_j,\theta , {\Lambda ^*_1},{\Lambda ^*_2})\right) ^2. \end{aligned}$$

Initial parameter values $\theta = \{0.5$ kPa, $12, 10, 15\}$ were uniformly applied to both control and SuHx tissues. Throughout the optimization, the model parameters were constrained to be positive with no set upper bounds. The resulting optimal parameter set was utilized to construct a strain-energy surface for each individual animals’ tissue sample, and these strain-energy surfaces were subsequently point-wise averaged within their respective groups. From the mean and standard error of the averaged strain-energy surfaces (Appendix Table 2), the equibiaxial stress–strain relations were determined. Anisotropy was quantified as the slope of the equibiaxial stress–strain curve (0.075$-$0.08 Green strain) and as the ratio of AOT-to-circumferential stress at 0.08 Green strain.

### Collagen assays

RV tissue adjacent to the biaxial sample was flash-frozen in liquid nitrogen, stored at -80°C, and used for collagen analysis. Total collagen content was measured via hydroxyproline using the *Sensitive Tissue Collagen Assay* (QuickZyme Biosciences, NL). Samples were hydrolyzed and incubated with assay reagents, after which absorbance at 560 nm (Tecan LifeSciences, CH) was measured. Collagen concentration was interpolated from a standard curve. Pyridinoline (PYD) cross-linking was assessed using a competitive ELISA kit (*Rat Pyridinoline ELISA Kit*, MyBioSource, CA, USA). Previously frozen RV samples were homogenized in cold PBS, sonicated, and centrifuged, and the resulting supernatants were assayed following manufacturer protocols. Absorbance at 450 nm was measured and converted to concentration using a best-fit standard curve. All collagen data were normalized to tissue weight then normalized by the group control to avoid the confounding effects of hypertrophy and growth.

### Statistical analysis

Hemodynamic, morphological, mechanical, and collagen data were analyzed using JMP Pro v17 (SAS Institute, NC, USA), with significance defined as $\alpha = 0.05$. Two-factor ANOVA was used for normally distributed data, with factors of experimental stage (Control, SuHx Week 4, SuHx Week 8, SuHx Week 12) and either sex (male, ovary-intact female) or group (male, ovary-intact female, OVX female). Significant effects were followed by Tukey post hoc comparisons. For nonparametric data, Wilcoxon/Kruskal-Wallis tests with Dunn’s post hoc tests were applied. The Wilcoxon Signed Rank test was used for paired analyses of the slopes of the equibiaxial stress–strain curves. Fold change analyses used Dunnett’s (parametric) or Dunn’s with control (nonparametric) tests. A Gaussian distribution was fit to the AOT stress: circumferential stress ratio distributions from 2:1 to 1:2.

Continuous stress–strain data were log-transformed (log(stress+1)) and modeled via a generalized linear mixed model. Significant interaction effects were found, so each group was analyzed separately:

9 $$\begin{aligned} {\textbf{D}}_{i} = \xi {\textbf{E}}_{i} + \kappa {\textbf{E}}_i + \beta + \epsilon _i \end{aligned}$$

where ${\textbf{D}}$ is the mean log-transformed (linear) stress, ${\textbf{E}}$ the Green strain sampled at each observation ($i = 1,..., n$), $\xi$ the group-specific slope of the $\mathbf {D-E}$ relation, $\kappa$ the experimental stage-specific slope adjustment factor, $\beta$ the experimental stage-specific intercept, and $\epsilon$ the random error term. All figures were generated using GraphPad Prism (version 10.3.1, GraphPad Software, MA, USA).

## Results

### Pulmonary hypertension alters RV hemodynamics

**Fig. 2:**
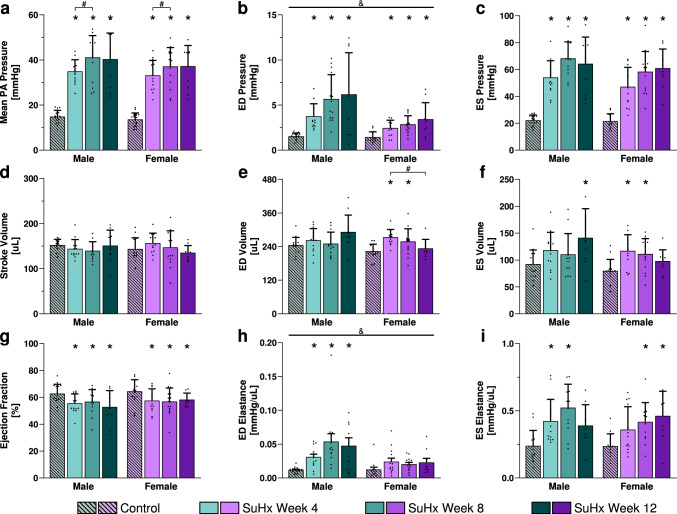
Right ventricular hemodynamic metrics measured in normotensive (control, patterned) and hypertensive (SuHx, solid) male (green) and ovary-intact female (purple) rats show significant differences in RV function. The most pronounced increases in mean pulmonary arterial pressure (**a)**, end-diastolic pressure (**b)**, and end-systolic pressure (**c)** occurred after four weeks of SuHx. Even though there were some increases in end-diastolic volume (**e)** and end-systolic volume (**f)** with SuHx, stroke volume (**d)** was maintained. Ejection fraction (**g)** decreased significantly by SuHx Week 4 in both groups but did not decrease more than $10\%$ by SuHx Week 12. Significant increases in end-diastolic elastance (**h)** occurred only in SuHx males, while end-systolic elastance (**i)** increased due to SuHx in both groups. Data are presented as mean ± standard error; $\star p < 0.05$ effect of SuHx compared to control, ${\texttt {\#}} p < 0.05$ effect of extended SuHx compared to earlier SuHx weeks, ${\texttt { \& }}p < 0.05$ difference between groups

At baseline, there were no differences found in right ventricular hemodynamic metrics between male and female rats. After four weeks of SuHx, there were significant increases in mPAP ($p < 0.001$), ED pressure (male: $p = 0.00201$; female: $p = 0.0391$), and ES pressure (male: $p = 0.00117$; female: $p = 0.00259$) in both groups (Fig. 2a–c). Mean pulmonary arterial pressure monotonically increased from $14.2\pm 0.5$ mmHg in normotensive controls to $34.1\pm 1.1$ mmHg in SuHx Week 4 rats, while ES pressure increased from $21.9\pm 0.8$ mmHg to $50.7\pm 2.6$ mmHg across the same time span. The increase in ED pressure by SuHx Week 4 was larger in male rats ($1.5\pm 0.1$ mmHg to $3.8\pm 0.4$ mmHg) than female rats ($1.4\pm 0.1$ mmHg to $2.5\pm 0.2$ mmHg).

Even though ED volume (Fig. 2e) increased significantly after four weeks of SuHx in females ($p < 0.001$), increases in ES volume (Fig. 2f, $p = 0.0103$) kept stroke volume (Fig. 2d) unchanged. Ejection fraction (Fig. 2g) significantly decreased ($p = 0.0104$), dropping over $8\%$ by SuHx Week 4, with no significant differences between groups. In male rats, SuHx Week 4 ED elastance (Fig. 2h) was more than 2.5-fold the normotensive diastolic elastance ($p = 0.0206$), while female ED elastance was not significantly elevated. By SuHx Week 4, ES elastance (Fig. 2i) was significantly higher than control in male ($p = 0.0199$) rats, but unchanged in females ($p = 0.388$).

An additional four weeks of SuHx resulted in another significant increase in mPAP ($p =0.0405$) to $39.1\pm 1.7$ mmHg (Fig. 2a). ED pressure (Fig. 2b) remained elevated in male and female rats at SuHx Week 8 ($5.7\pm 0.7$ mmHg and $2.9\pm 0.2$ mmHg, respectively). ES pressure (Fig. 2c) at SuHx Week 8 ($63.0\pm 2.6$ mmHg) was unchanged from SuHx Week 4 (male: $p = 0.531$; female: $p > 0.999$), but still significantly higher than control (both $p < 0.001$). Stroke volume continued to be maintained at around $150\mu$L, despite small changes in ED and ES volume (Fig. 2d–f). Although ejection fraction initially dropped at SuHx Week 4, no additional decrease was seen at SuHx Week 8 (Fig. 2g; $p > 0.999$). ED elastance (Fig. 2h) continued to only be significantly higher than control in males ($p < 0.001$), while ES elastance at SuHx Week 8 was significantly elevated from control in both groups (Fig. 2i; male: $p < 0.001$; female: $p = 0.0227$).

Hemodynamic metrics did not significantly change from SuHx Week 8 to SuHx Week 12. Pressures (Fig. 2a–c) stabilized, with mPAP, ED pressure, and ES pressure at SuHx Week 12 higher than control (mPAP: $p < 0.001$; ED pressure: male: $p = 0.00101$, female: $p = 0.00136$; ES pressure: $p < 0.001$), but not significantly different than SuHx Week 8 (all $p > 0.999$). Volumes (Fig. 2d–f) also largely stabilized. Although ejection fraction (Fig. 2g) was the lowest at SuHx Week 12 ($55.4\pm 2.1\%$, an $8\%$ drop), this value was only significantly different from the normotensive controls ($p = 0.00505$) and ejection fraction did not progressively decrease throughout the experimental timepoints (SuHx Week 12 compared to SuHx Week 4: $p = 0.972$).

### Organ level RV diastolic properties depend on ovarian hormones

**Fig. 3:**
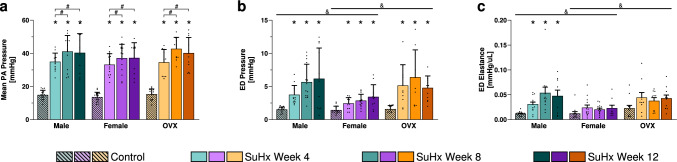
Right ventricular diastolic metrics measured in normotensive (control, patterned) and hypertensive (SuHx, solid) male (green), ovary-intact female (female, purple), and ovariectomized female (OVX, orange) rats show significant differences due to ovarian hormone presence. There was no difference in SuHx-induced mean pulmonary arterial pressure elevation (**a)** between groups. OVX end-diastolic pressures (**b)** increased due to SuHx and both male and OVX ED pressures were significantly higher than diastolic pressures in females. End-diastolic elastance (**c)** in OVX rats was elevated compared to ovary-intact females. Data are presented as mean ± standard error; $\star p < 0.05$ effect of SuHx compared to control, ${\texttt {\#}}p < 0.05$ effect of extended SuHx compared to earlier SuHx weeks, ${\texttt { \& }} p < 0.05$ difference between groups

Significant group differences between male and female hemodynamics were only seen in diastole. ED pressures and elastances in male rats were consistently higher, and showed greater increases with SuHx, than in female rats (Fig. 2b, h; ED pressure: $p = 0.0145$, ED elastance: $p = 0.0129$). Given that ovarian hormones such as estrogen are thought to influence RV function in PAH, ovariectomized female rats were included in the study to assess hormone-dependent effects. Hemodynamic metrics were measured in these animals alongside male and ovary-intact female rats to evaluate sex- and hormone-specific differences. No group differences were found in systolic properties, stroke volume, or ejection fraction (Appendix Fig. 10). Similarly, the increase in mPAP in OVX rats followed the same trend as in male and female rats, with no group differences (Fig. 3a). OVX ED pressures (Fig. 3b) were significantly increased in all SuHx weeks ($p < 0.001$), significantly higher than in female rats ($p = 0.0176$), and not significantly different from males ($p > 0.999$). At SuHx Week 4, ED pressures in OVX rats were significantly higher than ED pressures in female rats ($p = 0.0186$). By SuHx Week 8, both male ($p = 0.0120$) and OVX ($p = 0.0362$) ED pressures were significantly higher than females. While ED elastance in OVX SuHx rats (Fig. 3c) did not significantly increase ($p = 0.0846$) similar to the trend in female rats, diastolic elastances were higher in OVX rats than in females ($p = 0.00109$) and more comparable to ED elastance values in males ($p = 0.684$). Male SuHx Week 8 ED elastances were significantly higher than SuHx Week 8 female diastolic elastances ($p = 0.00333$). Appendix Fig. 9 illustrates the ovarian hormone-dependent differences in ED pressure and elastance between groups. The steady-state ED pressure was higher in male and OVX rats compared with ovary-intact females, highlighting group differences in diastole.

### Group and SuHx influence RV biaxial mechanical properties

Myocardium biaxial mechanical data were reproducible across successive tests on each sample and model fits were robust. Observed shear deformations were small, and no statistical difference was found between equibiaxial curves calculated using principal and normal strain analysis, allowing the use of normal strains and justifying ignoring shear deformation. Equibiaxial stress–strain relations were calculated up to 0.08 strain to ensure all the samples were mapped to a common Green-strain space.

**Fig. 4:**
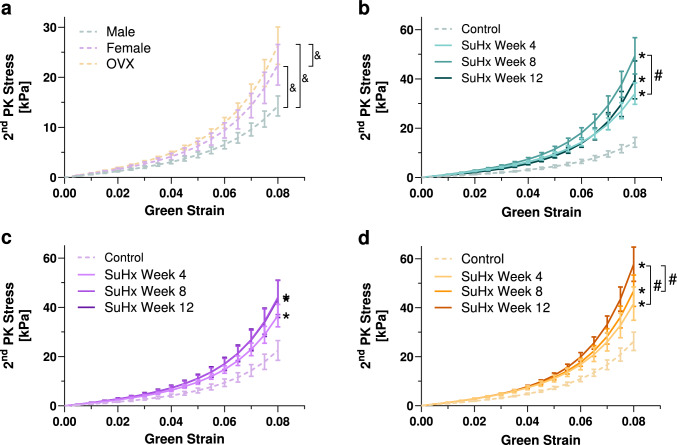
Apex-outflow tract direction stress–strain relations from normotensive (**a)** and hypertensive (**b**–**d)** male (green), ovary-intact female (purple), and ovariectomized female (orange) right ventricles show significant stiffening due to SuHx (darker colors represents later SuHx weeks). While normotensive male RV tissue is the most compliant (**a)**, it significantly stiffens in response to SuHx, peaking at SuHx Week 8 (**b)**. Female right ventricles (**c)** also significantly stiffen due to SuHx, with SuHx Weeks 8 and 12 closely overlapping. The OVX right ventricles are the stiffest in normotension and progressively stiffen with SuHx (**d)**. Data are shown as mean ± standard error; Green strain represents tissue deformation relative to the undeformed $\beta _0$ state; $\star p < 0.05$ effect of SuHx compared to control, ${\texttt {\#}} p < 0.05$ effect of extended SuHx compared to earlier SuHx weeks, ${\texttt { \& }} p < 0.05$ difference between groups

AOT stress–strain relations were significantly different across the normotensive control groups (Fig. 4a). Male RVs were significantly more compliant than either female or OVX RVs (male–female: $p = 0.00841$; male-OVX: $p < 0.001$) and within the two female sex groups, OVX rats had a stiffer myocardium than ovary-intact females ($p = 0.0158$). In all groups, four weeks of SuHx significantly increased RV stiffness ($p < 0.001$). After this initial SuHx-induced stiffening, the mechanical properties of ovary-intact female RVs were unchanged with additional SuHx (Fig. 4c; all $p > 0.566$). Male RVs (Fig. 4b) experienced additional significant stiffening by SuHx Week 8 ($p = 0.00264$), while OVX RVs (Fig. 4d) further stiffened by SuHx Week 12 ($p = 0.0262$).

**Fig. 5:**
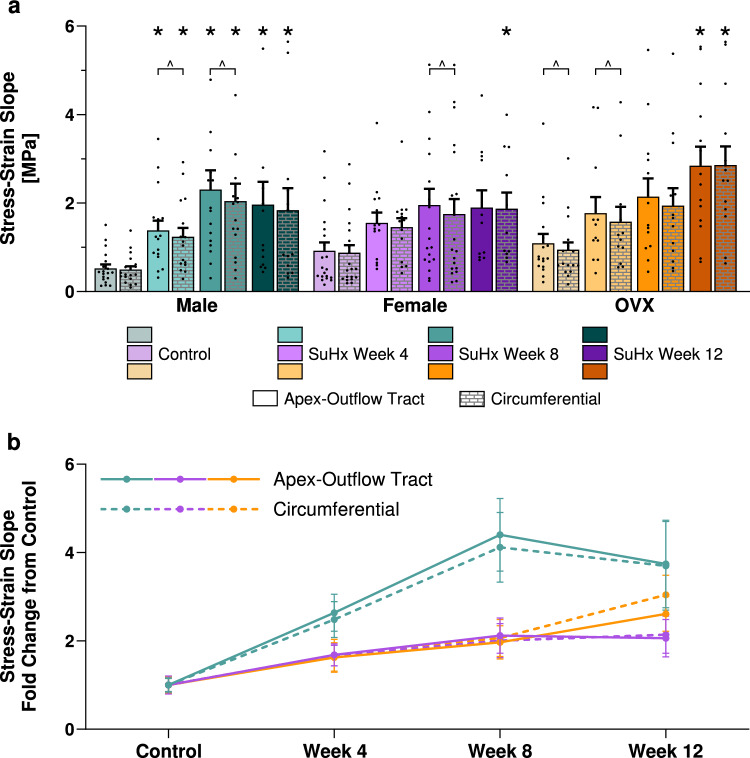
Stress–strain slopes of the right ventricular free wall under equibiaxial loading show stiffening due to SuHx is most predominant in male rats. Stress–strain slopes (**a)** in the AOT (solid) and circumferential (patterned) directions were greater than control in all male (green) SuHx weeks. Anisotropy was present in right ventricles from SuHx Week 4 and 8 males, SuHx Week 8 ovary-intact females (purple), and control and SuHx Week 4 ovariectomized females (OVX, orange). Fold change in stress–strain slope relative to normotensive controls (**b)** more than quadrupled from control by SuHx Week 8 in males, while in hypertensive ovary-intact female rats, the slope only doubled. OVX stress–strain slopes almost tripled by SuHx Week 12. Data are shown as mean ± standard error **a** and the mean ± standard error of the fold change **b**; $\star p < 0.05$ effect of SuHx compared to control, $\wedge p < 0.05$ difference between AOT and circumferential directions

Similar trends were observed in the stress–strain slopes (Fig. 5a). Normotensive OVX RVs exhibited higher slopes than normotensive males in both the AOT ($p = 0.0245$) and circumferential ($p = 0.0414$) directions. In males, stress–strain slopes increased at all SuHx weeks in both directions (all $p < 0.0203$). Ovary-intact female slopes increased only in the circumferential direction in SuHx Week 12 RVs ($p = 0.0398$), whereas SuHx Week 12 OVX RVs exhibited higher slopes in both directions (AOT: $p = 0.00238$; circumferential: $p < 0.001$).

As baseline stiffness (Fig. 4a) and the stress–strain slope (Fig. 5a) of the normotensive control RVs varied between groups, fold changes in slopes were calculated for both AOT and circumferential directions (Fig. 5b). At SuHx Week 4, stress–strain slopes increased more in males ($\sim 2.6\times$ control) than ovary-intact or OVX females ($\sim 1.7\times$ control). By SuHx Week 8, male slopes reached $\sim 4.3\times$ control, whereas both female groups’ slopes were $\sim 2.0\times$ their respective normotensive control. While OVX female stress–strain slopes further increased at SuHx Week 12 to $\sim 2.8\times$ control, ovary-intact female slopes plateaued after SuHx Week 8.

**Fig. 6:**
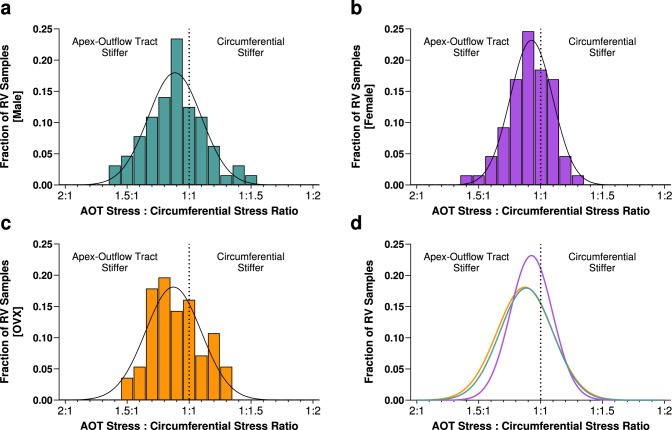
Anisotropy of RV tissue samples was biased toward the apex to outflow tract direction, although this varied on a tissue-to-tissue basis. Histograms of individual RV tissue stress ratios (apex to outflow tract stress to circumferential stress) are separated per animal group (**a**–**c)** and shown as the fraction of animals within each group. Gaussian curves (black solid lines) are fit to each distribution and isotropy is represented with a vertical black dashed line. The Gaussian distributions for male (green), ovary-intact female (purple), and ovariectomized female (OVX, orange) are overlaid for comparison (**d)**

Although only AOT direction stress–strain relations are shown here, the circumferential axis followed the same trends in all groups and experimental stages. Anisotropy was assessed using both the stress–strain slopes (Fig. 5) and the AOT-to-circumferential stress ratio (Fig. 6). Rat-specific AOT:circumferential stress ratios ranged from 1.6:1 to 1:1.5 (Fig. 6a–c). Gaussian fits of each group’s distribution (Fig. 6d) showed overlapping 95% confidence intervals (Male: 1.12:1 [1.15:1, 1.08:1], Female: 1.07:1 [1.09:1, 1.06:1], OVX: 1.13:1 [1.17:1, 1.08:1]), indicating no significant differences in anisotropy between groups.

Statistical comparisons of the stress–strain slopes (Fig. 5a) confirmed that male and ovary-intact female control RVs were isotropic (male: $p = 0.515$, female: $p = 0.0897$), whereas OVX controls were stiffer in the AOT direction ($p = 0.00316$). At SuHx Week 4, AOT slopes were greater than circumferential slopes in both male ($p = 0.0214$) and OVX ($p = 0.0425$) RVs. Male anisotropy persisted at SuHx Week 8 ($p = 0.0131$), while OVX RVs became isotropic ($p = 0.0681$). Ovary-intact female RVs were only anisotropic, with the AOT direction being stiffer, at SuHx Week 8 ($p < 0.001$).

### Changes in RV wall hypertrophy are sex dependent

**Fig. 7:**
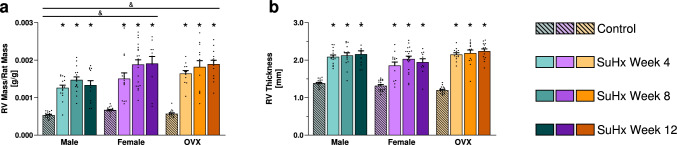
Marked increase in right ventricle hypertrophy due to wall thickening occurs by SuHx Week 4 in all animal groups. Right ventricular mass normalized by rat body mass (**a)** increased significantly with four weeks of SuHx, then stabilized through SuHx Week 12. Greater increases in normalized right ventricle mass are seen in ovary-intact (female, purple) and ovariectomized (OVX, orange) females compared to males (green). Right ventricular wall thickness (**b)** increased significantly in all groups after four weeks of SuHx but did not continue to increase in later SuHx weeks. Data are presented as mean ± standard error; $\star p < 0.05$ effect of SuHx compared to control, ${\texttt {\#}} p < 0.05$ effect of extended SuHx compared to earlier SuHx weeks, ${\texttt { \& }} p < 0.05$ difference between groups

To account for growth across the experimental stages and different growth rates between animals in each group, RV mass was normalized by rat body mass (Fig. 7a). Normalized RV mass significantly increased by SuHx Week 4 ($p < 0.001$). No additional hypertrophy was seen between SuHx Weeks 4 and 12 ($p > 0.999$); however, normalized RV mass remained elevated in all groups. Normalized RV mass was significantly greater in female and OVX RVs compared to males (male–female: $p = 0.0113$; male-OVX: $p = 0.0371$). RV tissue thickness (Fig. 7b) increased significantly by SuHx Week 4 (male: $p < 0.001$; female: $p = 0.00198$; OVX: $p < 0.001$), but no further thickening was seen with additional SuHx, and there were no differences between groups ($p = 0.109$).

### Ovarian hormones influence collagen cross-linking, but not accumulation

**Fig. 8:**
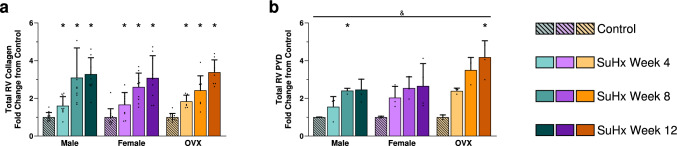
Collagen accumulates in the RV by SuHx Week 8 in all animal groups, but enzymatic cross-linking increases the most in ovariectomized (OVX, orange) female rats. Total collagen content measured by hydroxyproline and normalized to group controls (**a)** increased significantly after eight weeks of SuHx, with the largest increase in fold change seen in males between SuHx Week 4 and SuHx Week 8. Total pyridinoline (PYD) cross-link concentration normalized to group controls (**b)** was only statistically significantly increased in male SuHx Week 8 and OVX SuHx Week 12, with a greater overall increase seen in OVX rats. Data are presented as mean ± standard error of the fold change relative to control; $\star p < 0.05$ effect of SuHx compared to control, ${\texttt { \& }} p < 0.05$ difference between groups

Total collagen content measured by hydroxyproline was normalized by each group’s control to account for differences in hypertrophy across the experimental stages (Fig. 8a). Total collagen was significantly elevated from control by SuHx Week 8 ($p < 0.001$) in all groups. By SuHx Week 12, RVs from male, female, and OVX rats had over three times as much collagen as RVs from their respective control groups. No differences in collagen accumulations were seen between groups ($p = 0.862$). Total pyridinoline cross-links were also normalized by group controls (Fig. 8b). Although enzymatic cross-linking increased to over $2.5\times$ control in females, this change was not significant ($p = 0.0670$). Cross-linking was only significantly elevated from control in male SuHx Week 8 ($p = 0.0488$) and OVX SuHx Week 12 ($p = 0.0102$), although overall, OVX RVs had more enzymatic cross-links than males ($p = 0.0175$).

## Discussion

This study examined how sex and ovarian hormones modulate right ventricular remodeling across four stages of pulmonary hypertension in male, ovary-intact female, and ovariectomized female rats. Significant ovarian hormone-dependent differences were found in diastole; increases in ED pressure, elastance, and passive myocardial stiffness were attenuated in hypertensive rats with normal levels of circulating ovarian hormones. We first evaluated baseline sex differences under normotensive conditions. Organ-level hemodynamics were similar between groups; however, tissue-level stiffness differed. Normotensive male myocardium was significantly more compliant than both female groups’ myocardium, while OVX myocardium was stiffest. To our knowledge, this is the first report of baseline sex-dependent RV myocardial stiffness. No group differences were found in total collagen or pyridinoline cross-link concentration in normotensive rats.

Despite similar baseline characteristics and exposure to comparable systolic pressure overload, RV remodeling diverged markedly between groups. Diastolic remodeling was strongly influenced by ovarian hormone status. In males and OVX females with low-to-no circulating ovarian hormones, fold-change increases in ED pressure matched or exceeded increases in systolic pressure at each experimental stage. Although ED pressure was also significantly higher than control in hypertensive ovary-intact females, the elevation in diastolic pressure was much less severe than the increase in systolic pressure. By SuHx Week 12, ED pressure in ovary-intact females remained lower than that of SuHx Week 4 male and OVX rats, suggesting ovarian hormones mitigated diastolic dysfunction. Ovary-intact females also showed lower diastolic chamber elastances and the smallest peak myocardial stress, consistent with previous work that implicated estrogen in protecting against adverse remodeling (Frump et al. 2015). Sex also played a role in RV remodeling, as neither female group exhibited a significant increase in ED elastance with SuHx and tissue-level stiffening was approximately half that observed in males. Despite differential stiffening between groups, similar collagen accumulation was observed in all RVs. This finding echoes the conclusion of Kakaletsis et al. (2023) that collagen content alone does not account for increased myocardial stiffness. Collagen cross-linking may be key in the remodeling process, as only male and OVX rats showed significant increases in pyridinoline cross-links, correlating with the most pronounced stiffening after the initial remodeling to SuHx Week 4 and to the stiffest RVs.

### RV remodeling in males

Four weeks of SuHx gave rise to pulmonary hypertension and robust RV remodeling in male rats, consistent with previous findings (Kwan et al. 2021; Legchenko et al. 2018; Mendiola et al. 2023; Neelakantan et al. 2025; Toba et al. 2014). Both systolic and diastolic pressures rose significantly, with a comparably larger fold-change increase in ED pressure. As with previous longitudinal investigations, pulmonary and ventricular pressures initially increased before stabilizing at later SuHx weeks (Jayasekera et al. 2020; Kwan et al. 2021). This trend was also seen with hypertrophy and tissue stiffening, indicating that substantial RV remodeling occurred rapidly in response to SuHx with more mild additional alterations needed to maintain RV function over time.

Importantly, volumes remained stable despite pressure increases, indicating preserved chamber filling. The increase in ED pressure was matched with a similar fold-change increase in ED elastance, an established prognostic indicator of PAH severity (Rain et al. 2013; Trip et al. 2015; Vanderpool et al. 2022). Kwan et al. (2024) previously showed that ED elastance was significantly elevated in male SuHx Week 8 rats, and their model predicted that males would undergo the most severe myocardial passive stiffening. We observed a similar significant increase in ED elastance in male SuHx rats and were able to confirm RV passive stiffening at the tissue level through ex vivo biaxial mechanical tests. Prior studies have documented pressure overload-induced stiffening at single timepoints. (Avazmohammadi et al. 2017; Hill et al. 2014; Jang et al. 2017; Kakaletsis et al. 2023; Neelakantan et al. 2025; Park et al. 2016; Vélez-Rendón et al. 2019); this study demonstrates that stiffening progresses with prolonged SuHx exposure.

Rapid RV hypertrophy is a well-characterized response to pressure overload in animal models and in patients with pulmonary hypertension (Abe et al. 2010; Andersen et al. 2014; Avazmohammadi et al. 2019; Baicu et al. 2012; Gan et al. 2007; van Wezenbeek et al. 2022; Vélez-Rendón et al. 2018). Here, hypertrophy initially stemmed from increased wall thickness and later was likely driven by collagen accumulation, consistent with remodeling patterns observed in the pulmonary artery banding model (Baicu et al. 2012). Variability in collagen content assessment across studies may stem from differences in timing, measurement site, and collagen quality (e.g., cross-linking), which our findings suggest play a critical role in functional remodeling.

### RV remodeling in females

Although PAH incidence and clinical outcomes are related to sex, previous longitudinal time-course studies of pulmonary hypertension in animal models have been conducted with only male subjects (Gerringer et al. 2018; Jayasekera et al. 2020; Kwan et al. 2021). To our knowledge, this is the first study to investigate progressive RV remodeling due to pulmonary hypertension in a female animal model and show that ovary-intact female RVs undergo less extreme diastolic changes in response to pressure overload than males. The fold-change increase in ES pressure in ovary-intact females was significantly greater than the fold-change increase in ED pressure, in direct contrast to the trend observed in males and OVX females where ED pressure increased more rapidly. Using the pulmonary artery banding model of pressure overload, Cheng et al. (2022) found similar increases in mPAP and ES pressure with our study, but conflicting ED pressure measurements. In their rats, ED pressure was only significantly elevated from sham in ovary-intact females, with smaller increases observed in males and ovariectomized females. The more rapid onset of pressure overload in the pulmonary artery banding model compared to the SuHx model is the most likely cause of the ED pressure discrepancies; however, both studies show the importance of ovarian hormones in regulating diastolic RV remodeling as ovary-intact and OVX females behaved differently.

The loss of ovarian hormones in menopause is linked to an increased risk of cardiovascular disease in women (Conway-O’Donnell and Chesler 2022; Zhao et al. 2014). Many studies using female animals investigate the effect of endogenous or exogenous estrogen by comparing ovary-intact females to ovariectomized females with or without estrogen repletion. In agreement with our results, several of these studies show that SuHx results in larger increases in ES pressure and RV wall thickness in the ovariectomized group with no exogenous estrogen (Frump et al. 2015; Lahm et al. 2016; Liu et al. 2017), although most of these differences are not statistically significant. Estrogen deficiency also activates the renin–angiotensin–aldosterone system (RAAS) and increases cardiac angiotensin II (Chappell et al. 2003; Zhao et al. 2014), decreases tetrahydrobiopterin ($BH_4$) and alters nitric oxide synthase (NOS) activity, increasing reactive oxygen species (ROS) (Zhao et al. 2014), reduces estrogen receptor $\alpha$ (ER$\alpha$) expression (Frump et al. 2015, 2021), and impairs mitochondrial density and oxidative capacity (Liu et al. 2017). Estrogen replacement mitigates these effects, supporting ovarian hormone-dependent modulation of RV function.

Using the SuHx rat model at a single timepoint, Kwan et al. (2024)’s study matched our systolic function in female SuHx rats but showed no ovarian hormone-dependent diastolic hemodynamic properties. However, their model did show that the increase in ED pressure in ovary-intact female RVs was almost entirely explained by geometric remodeling, while male and ovariectomized female rats required myocardial stiffening to reach SuHx ED pressures. Additionally, their model predicted that both groups of female rats would undergo less passive RV stiffening than the male rats, which was confirmed by our biaxial mechanical data. Witzenburg et al. (2012) mechanically tested RV samples from normotensive female rats and found the tissue to be very anisotropic with the circumferential direction being stiffest. This contrasts with our data which showed that both normotensive and hypertensive RVs were marginally stiffer in the AOT direction. These differences are most likely due to variations in the sample orientation and significant differences in the mechanical testing protocols.

As with studies in male rats, there is variability in RV collagen assessment in females, although most studies agree that collagen fraction is increased in ovariectomized rats compared to their ovary-intact counterparts (Cheng et al. 2022; Lahm et al. 2016). In our study, pyridinoline enzymatic cross-links were most prevalent in ovariectomized SuHx rats, and others have shown that the collagen type I:III ratio (Lahm et al. 2016) and fiber thick:thin ratio (Cheng et al. 2022) were increased in ovariectomized rat RVs. Together, this suggests that structural changes to the collagen extracellular matrix, rather than collagen accumulation, may explain some of the observed ovarian hormone-dependent differences in diastolic properties. Although collagen cross-linking was similar in males and females, their stiffening responses differed, suggesting that additional contributors—such as myocyte stiffness, connectivity, and alignment—play an important role alongside extracellular matrix remodeling.

### Diastolic RV function in PAH patients

Although many clinical studies include both men and women, few report sex-stratified RV metrics, particularly those focused on diastolic RV function (Gan et al. 2007; Rain et al. 2013; Trip et al. 2015; Vanderpool et al. 2022). Furthermore, little information is available regarding hormone levels or menopause status; at best some studies stratify the female cohort by age (van Wezenbeek et al. 2022) in an attempt to separate women of reproductive age from those in menopause. In accordance with our results, most patient studies that separate men and women found no sex differences in mPAP (Jacobs et al. 2014; Kozu et al. 2018; van Wezenbeek et al. 2022; Ventetuolo et al. 2014, 2017). Our measured hemodynamic values from open-chest surgery tended to be lower than those measured in patients with pulmonary hypertension through closed-chest right heart catheterization, though the trends were largely the same (Gan et al. 2007; Jacobs et al. 2014; Kozu et al. 2018; Trip et al. 2015; van Wezenbeek et al. 2022; Vanderpool et al. 2015; Ventetuolo et al. 2017). The notable exception was ejection fraction which stabilized around $55\%$ in our SuHx rats, higher than typical PAH patients, but consistent with previous studies using animal models (Kwan et al. 2024, 2021; Vélez-Rendón et al. 2018).

Diastolic dysfunction, and in particular diastolic stiffness, has increasingly been associated with clinical outcomes in patients (Gan et al. 2007; Rain et al. 2013; Trip et al. 2015). Although we only observed significantly elevated end-diastolic elastance in our male SuHx rats, all three groups experienced significant tissue-level passive myocardial stiffening: Peak stress approximately doubled in females and almost quadrupled in males. In patients, higher ED elastance correlates with lower ejection fraction and worse survival (Trip et al. 2015). As tissue-level stiffness can’t be measured clinically, echocardiographic surrogates such as the ratio between the lateral tricuspid annulus peak systolic velocity ($S'$) and the right atrial area indexed to body surface area (*RAAi*) have been proposed (Yogeswaran et al. 2023) to aid in assessing RV diastolic dysfunction. This metric ($S'/RAAi$) moderately correlates with both ED pressure and ED elastance and has been shown to outperform tricuspid annular plane systolic excursion (TAPSE) in predicting poor outcomes in PAH.

### Limitations

This study relied on separate groups of animals at each experimental stage, rather than tracking the same individuals over time. As a result, some temporal trends—such as the apparent reduction in RV stiffness observed in SuHx Week 12 males—may reflect inter-animal variability rather than a reversal in remodeling. Future studies incorporating noninvasive imaging could enable within-subject tracking and more definitive assessments of RV progression. Ovarian hormone levels were not directly measured; however, ovariectomy in the OVX female rats was verified at terminal surgery by excising the uterus and confirming the absence of ovaries. Additionally, the rats included in this study were relatively young, which limits direct comparisons of the ovary-intact female and OVX female rats to pre- and postmenopausal women, respectively. We prioritized standardizing the age and weight of the rats at the time of SuHx administration, eliminating the effect of aging and focusing solely on the presence or absence of ovarian hormones.

Although diastolic right ventricular stretches can exceed $20\%$ and anisotropy generally increases at higher strains, hypertensive RVs typically exhibit longitudinal strain below $10\%$ (Mendiola et al. 2023). To characterize passive SuHx RV myocardium while minimizing tissue damage and preserving collage architecture, experimental stretches were limited to $10\%$. As a result, anisotropy measured in this study may underestimate the full mechanical differences that could emerge at higher physiological strains. Although tissue thickness—and thus mass—varied between samples, the biaxial preload was not mass normalized. This may have caused differences in the initial stretch; however, preloading resulted in minimal deformation and non-planar displacement remained negligible throughout testing, mitigating the potential impact of the initial stretch on the results. Another limitation is the spatial specificity of mechanical testing. Biaxial tests were performed on a standardized square sample from the RV free-wall to ensure consistency across animals. However, this approach inherently excludes regional heterogeneity, including differences near the septum or atrium. Due to the small size of the rat RV, collecting multiple mechanical datasets from the same heart is not feasible. Additionally, although we prioritized aligning mechanical and biochemical analyses by sampling adjacent tissue for collagen assays, this strategy limited our ability to assess whole-RV collagen distribution.

## Conclusion and significance

This study demonstrates that sex and ovarian hormones influence the progression and severity of RV remodeling during pulmonary hypertension. While all groups exhibited pressure-induced changes in chamber structure and function, ovary-intact females experienced milder diastolic dysfunction and less myocardial stiffening than male or OVX rats. These differences were not explained by total collagen content but were associated with elevated collagen cross-linking in groups lacking ovarian hormones. Our findings suggest that the presence of ovarian hormones helps limit the extent of SuHx-induced RV remodeling. These results underscore the importance of including both sexes and hormonal status in preclinical models of PAH and point to extracellular matrix cross-linking as a potential therapeutic target in right ventricular dysfunction. Future work should assess whether the baseline sex differences in right ventricular myocardia stiffness observed in Sprague Dawley rats are conserved in other animal models and in humans. Additionally, future studies should explore whether patient-derived metrics such as ED elastance or $S'/RAAi$ capture the sex and ovarian hormone-specific patterns we observed in tissue biomechanics and overall diastolic function.

## Figures and Tables

**Fig. 9:**
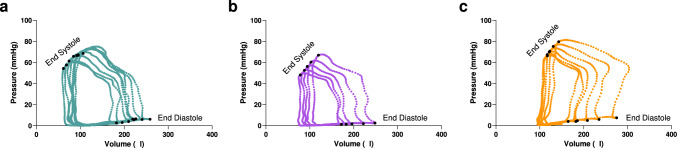
Representative pressure–volume loops recorded during caval occlusion in male (**a**, green), ovary-intact female (**b**, purple), and ovariectomized female (**c**, orange) SuHx Week 8 rats, where end systole and end diastole points are marked in black

**Fig. 10:**
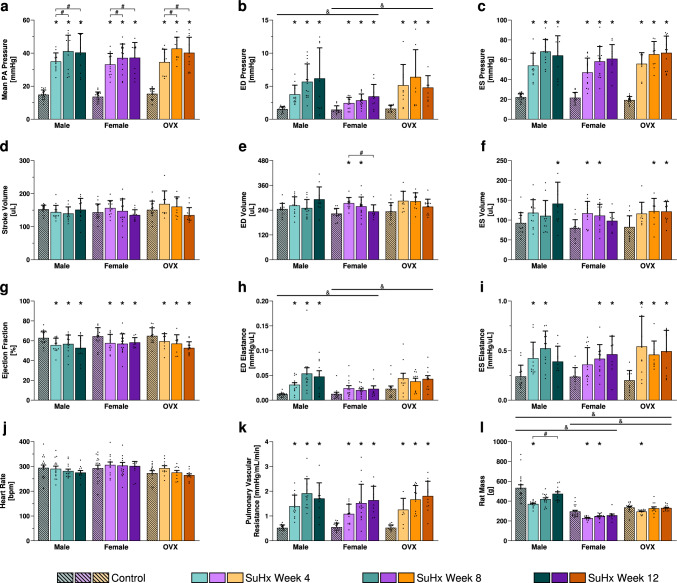
Right ventricular hemodynamic and morphological metrics measured in normotensive (control, patterned) and hypertensive (SuHx, solid) male (green), ovary-intact female (purple), and ovariectomized female (OVX, orange) rats show significant differences in RV diastolic function and body mass. Mean pulmonary arterial pressure (**a**), end-systolic pressure (**c**), end-diastolic volume (**e**), end-systolic volume (**f**), end-systolic elastance (**i**), and pulmonary vascular resistance (**k**) increased similarly in all groups with SuHx. Stroke volume (**d**) and heart rate (**j**) were maintained throughout. Ejection fraction (**g**) decreased in all groups by SuHx Week 4, but did not drop below $50\%$ at any timepoint. The most pronounced elevations in end-diastolic pressure (**b**) were seen in male and OVX SuHx rats in conjunction with significantly higher end-diastolic elastances (**h**). Rat body mass (**l**) was largest in males and smallest in ovary-intact females. Data are presented as mean ± standard error; $\star p < 0.05$ effect of SuHx compared to control, ${\texttt{\#}} p < 0.05$ effect of extended SuHx compared to earlier SuHx weeks, $\texttt { \& }$ $p < 0.05$ difference between groups

## Data Availability

Data will be made available upon reasonable request.

## Appendix

See (Figs. 9, 10 and Table 2).

**Table 2:** Model parameters of the averaged strain-energy surface for each group

Group	*C* [kPa]	$\hat{a}_1$	$\hat{a}_2$	$\hat{a}_3$
Male Control	0.379 [0.330, 0.432]	13.8 [12.3, 14.8]	12.8 [12.2, 13.2]	10.1 [9.66, 10.4]
Male SuHx Week 4	0.617 [0.563, 0.682]	17.2 [15.8, 18.2]	15.0 [12.8, 16.5]	11.2 [10.9, 11.2]
Male SuHx Week 8	0.541 [0.505, 0.590]	21.4 [19.6, 22.5]	18.4 [16.4, 19.7]	13.7 [12.3, 14.7]
Male SuHx Week 12	0.394 [0.457, 0.378]	21.8 [16.8, 25.3]	19.3 [13.9, 23.1]	14.4 [12.9, 14.9]
Female Control	0.409 [0.319, 0.503]	16.2 [15.0, 16.8]	15.0 [14.2, 15.5]	12.6 [11.5, 13.2]
Female SuHx Week 4	0.567 [0.525, 0.618]	17.9 [16.7, 18.6]	16.4 [15.3, 17.1]	12.7 [11.3, 13.8]
Female SuHx Week 8	0.587 [0.467, 0.709]	18.8 [18.7, 18.8]	15.8 [15.1, 16.2]	14.7 [13.1, 15.7]
Female SuHx Week 12	0.653 [0.470, 0.837]	16.9 [17.2, 16.6]	17.0 [17.2, 16.9]	14.2 [12.7, 15.0]
OVX Control	0.460 [0.497, 0.450]	16.9 [13.9, 19.2]	13.9 [11.8, 15.3]	12.8 [11.1, 13.9]
OVX SuHx Week 4	0.689 [0.673, 0.727]	17.8 [15.6, 19.4]	15.8 [14.1, 16.9]	12.7 [11.0, 13.8]
OVX SuHx Week 8	0.588 [0.574, 0.614]	20.1 [17.9, 21.8]	17.1 [15.3, 18.5]	13.2 [12.6, 13.6]
OVX SuHx Week 12	0.533 [0.503, 0.570]	21.4 [19.9, 22.5]	21.8 [20.3, 22.9]	15.5 [14.8, 16.0]

Parameters are presented as the optimized parameter value with the corresponding 95% confidence interval
